# Dicer-Like Genes Are Required for H_2_O_2_ and KCl Stress Responses, Pathogenicity and Small RNA Generation in *Valsa mali*

**DOI:** 10.3389/fmicb.2017.01166

**Published:** 2017-06-23

**Authors:** Hao Feng, Ming Xu, Yangyang Liu, Ruqing Dong, Xiaoning Gao, Lili Huang

**Affiliations:** College of Plant Protection and State Key Laboratory of Crop Stress Biology for Arid Areas, Northwest A&F UniversityYangling, China

**Keywords:** Dicer, pathogenesis, RNAi mechanism, small RNA, *Valsa mali*

## Abstract

*Valsa mali* (*V. mali*) is the causative agent of apple tree *Valsa* canker, which heavily damages the production of apples in China. However, the biological roles of the RNA interfering (RNAi) pathway in the pathogenicity of *V. mali* remain unknown. Dicer-like proteins (DCLs) are important components that control the initiation of the RNAi pathway. In this study, *VmDCL1* and *VmDCL2* were isolated and functionally characterized in *V. mali*. VmDCL1 and VmDCL2 are orthologous in evolution to the DCLs in *Cryphonectria parasitica*. The deletion of *VmDCL1* and *VmDCL2* did not affect vegetative growth when the mutants (Δ*VmDCL1*, Δ*VmDCL2* and Δ*VmDCL1DCL2*) and wild type strain 03–8 were grown on a PDA medium at 25°C in the dark. However, the colony of Δ*VmDCL1* increased by 37.1% compared to the 03–8 colony in a medium containing 0.05% H_2_O_2_ 3 days after inoculation, and the growth of Δ*VmDCL1* was significantly inhibited in a medium containing 0.5 M KCl at a ratio of 25.7%. Meanwhile, in the presence of 0.05% H_2_O_2_, the growth of Δ*VmDCL2* decreased by 34.5% compared with the growth of 03–8, but Δ*VmDCL2* grew normally in the presence of 0.5 M KCl. More importantly, the expression of *VmDCL2* was up-regulated 125-fold during the pathogen infection. In the infection assays using apple twigs, the pathogenicity of Δ*VmDCL2* and Δ*VmDCL1DCL2* was significantly reduced compared with that of 03–8 at a ratio of 24.7 and 41.3%, respectively. All defective phenotypes could be nearly rescued by re-introducing the wild type *VmDCL1* and *VmDCL2* alleles. Furthermore, the number and length distribution of unique small RNAs (unisRNAs) in the mutants and 03–8 were analyzed using deep sequencing. The number of unisRNAs was obviously lower in Δ*VmDCL1*, Δ*VmDCL2* and Δ*VmDCL1DCL2* than that in 03–8, and the length distribution of the sRNAs also markedly changed after the *VmDCLs* were deleted. These results indicated that *VmDCLs* function in the H_2_O_2_ and KCl stress response, pathogenicity and generation of sRNAs.

## Introduction

RNA interference (RNAi) is a gene silencing mechanism at the post-transcriptional level. It is triggered by the presence of double-stranded RNA molecules that are homologous to the corresponding target gene, thereby resulting in the degradation of the messenger RNA and attenuation of the target gene expression (Pratt and MacRae, 2009). This mechanism was first observed in petunia plants; in an attempt to over-express the enzyme chalcone synthase, the plants showed a purple pigment (Napoli et al., 1990). This process was called post-transcriptional gene silencing. The first study to report RNA silencing in fungi was performed in *Neurospora crassa*, and alterations of albino gene resulted in an albino phenotype (Romano and Macino, 1992). This phenomenon was termed “quelling." Subsequently, Fire et al. (1998) found RNA mediated genetic interference in *Caenorhabditis elegans*. This phenomenon was named RNAi. However, RNAi in animals is equivalent to post-transcriptional gene silencing in plants or gene quelling in fungi since they all work using a series of basic components, such as Dicer, Argonaute and RNA-dependent RNA polymerase (Fagard et al., 2000; Ipsaro and Joshua-Tor, 2015; Armas-Tizapantzi and Montiel-González, 2016). In the RNAi pathway, Dicer cleaves long dsRNA into short RNA duplexes of approximately 21–25 nucleotide (nt) small non-coding RNAs (sRNAs) (Zamore et al., 2000; Bernstein et al., 2001; Chapman and Carrington, 2007). Subsequently, these sRNAs are incorporated into the RISC to target the corresponding cognate mRNA for its destruction or translational suppression as sequence-specific guides (Hammond et al., 2000; Kim et al., 2010). Therefore, Dicer protein-mediated RNA cleavage is the initiation step of RNAi, and Dicer is essential for the generation and action of sRNAs.

Dicer or Dicer-like (DCL) protein is an endonuclease belonging to the RNase III family and contains a typical structure with an RNA helicase domain and a PAZ domain at the N′ terminal and two RNase III domains and a double-stranded RNA binding domain (dsRBD) at the C terminal (MacRae et al., 2006). Genome-wide sequencing projects have revealed that DCLs are evolutionarily conserved in a wide range of eukaryotic genomes (Kadotani et al., 2004). The fungal kingdom comprises an enormous diverse group of organisms, and the structure and number of DCLs also showed a conserved evolution among different species of fungi (Nakayashiki et al., 2006; Nunes et al., 2011b). However, the biological functions of fungal DCLs are diverse among different species. There have been some reports regarding the function of Dicers in sRNA generation. In 2004, *dcl-2* was shown to be indispensable in the siRNA generation process in *Magnaporthe grisea* (*Magnaporthe oryzae*) (Kadotani et al., 2004). In *N. crassa*, the generation of siRNA was not markedly affected in the single-knockout mutant of *Dicer-1* and *Dicer-2* but was abolished in the double-knockout mutant (Catalanotto et al., 2004). Knocking out Dicer genes could also affect the generation and accumulation of sRNAs in *Mucor circinelloides* (Nicolás et al., 2007, 2010; de Haro et al., 2009). Additionally, fungal *Dicers* have been shown to be associated with growth, development, environmental stress and pathogenicity. In *Mucor circinelloides*, the silencing of *Dicers* caused altered phenotypes, such as changes in morphology, growth rate, asexual sporulation and autolysis (Nicolás et al., 2007, 2015). Knocking out the *DCLs* in *Trichoderma atroviride* also resulted in significant changes in morphology, growth rate and sporulation (Carreras-Villaseñor et al., 2013). Segers et al. (2007) found that the interruption of the *dc1-1* and *dcl-2* genes in *Cryphonectria parasitica* produces strains that are highly susceptible to hypovirus infection, which was the first report regarding the role of fungal immunity against a virus. In *Magnaporthe oryzae*, only a growth inhibition was found in the *Modcl2* mutant, and the pathogenicity of the *Modcl1, Modcl2* and *Modcl1dcl2* mutants was not affected (Kadotani et al., 2004). Meanwhile, the virulence of *Sporisorium reilianum DCL* mutants was also not significantly changed (Schirawski et al., 2010). More importantly, when both Dicer genes were double-knocked out in *Botrytis cinerea*, the mutants showed a weakened pathogenicity (Weiberg et al., 2013). These results suggest that the fungal *Dicers* play important and various roles in specific physiological, developmental, and pathogenic processes. In-depth studies investigating the functions of the Dicer genes will lay the foundation for the exploration of the RNAi mechanism of fungi.

*Valsa mali* (*V. mali*) is one of the most important plant pathogens, causing canker disease in apple trees, which severely restricts the development of the apple industry and has been the key target of prevention and treatment in apple production in China (Cao et al., 2009; Wang et al., 2014). The lack of information regarding the molecular pathogenic mechanism in this fungus is the primary limitation in disease management. Therefore, the exploration of the pathogenic mechanism of *V. mali* has become extremely urgent. *V. mali* has a relatively small genome that was recently sequenced, and the major components of the RNAi pathway, such as Dicer, AGO, and RdRP, have been isolated (Yin et al., 2015). More importantly, most of these components exhibit regulated expression profiles during the *V. mali* infection process (Ke et al., 2014). In this study, we demonstrated that the Dicer genes of *V. mali* were responsible for the growth, development, and pathogenic processes and sRNAs generation based on gene knock-out mutants and high-throughput sequencing. This study laid a foundation for the comprehensive exploration of the pathogenesis mechanism of *V. mali*.

## Materials and Methods

### Fungus Strains and Growth Conditions

The *V. mali* wide type strain 03–8 used in this study was deposited in the Laboratory of Pathogen Biology and Integrated Control of Fruit Tree Diseases, College of Plant Protection, Northwest A&F University, Yangling, Shaanxi, China. The wild type and mutant strains generated in this study were maintained on a potato dextrose agar (PDA, 20% potato, 2% glucose, and 1.5% agar) medium at 25°C in the dark. Liquid potato dextrose broth (PDB) was used as the growth medium in the mycelium collection.

### Sequence Characterization and Phylogenetic Analysis

The *VmDCL* sequences were isolated from the available *V. mali* genome database using the *DCL* sequences of *M. oryzae* and *N. crassa* as the probe sequences for the *in silico* cloning. The domain architecture was analyzed using InterProScan^1^. A phylogenetic comparison of the deduced protein sequences and the corresponding proteins from other species was performed with DNAMAN version 6.0.

### Nucleic Acid Isolation and Manipulation

The fungal genomic DNA was extracted using the CTAB method (Möller et al., 1992). For the RNA isolation, apple twigs infected with *V. mali* strain 03–8 were sampled at 48 hpi (hours post inoculation). RNA samples from 03–8 mycelium and apple twigs infected by 03–8 were isolated using the TRIzol^TM^ Reagent (Invitrogen, Carlsbad, CA, United States) according to the manufacturer’s instructions. Contaminated genomic DNA was removed using DNase I (Invitrogen, Carlsbad, CA, United States). DNA/RNA degradation was monitored on 1% agarose gels. A NanoDrop^TM^ 1000 spectrophotometer (Thermo Fisher Scientific, United States) was used to assess the DNA/RNA purity, concentration and integrity.

### cDNA Synthesis and qRT-PCR Analysis

In total, three micrograms of total RNA were used to synthesize the first-strand cDNA using an RT-PCR system (Thermo Fisher Scientific, United States) according to the manufacturer’s instructions. The quantitative PCR amplifications were performed on a CFX96 Real-Time System (Bio-Rad) with SYBR Green (Invitrogen). The Glyceraldehyde-6-phosphate dehydrogenase gene (G6PDH) of *V. mali* was used as the internal control gene (Yin et al., 2013). The relative expression levels of each gene were calculated by the 2^-ΔΔCT^ method (Livak and Schmittgen, 2001). Three biological replicates were performed for each experiment. All primers are listed in Supplementary Table S1.

### Gene Deletion and Complementation

The vector pBIG2RHPH2-GFP-GUS, which contains a hygromycin resistance gene, was used to construct the gene deletion vector. The double-joint PCR approach was used for the vector construction and generating the gene knock-out mutants (Yu et al., 2004). For the single gene knock-out mutants, the strategy consisted of replacing the *VmDCLs* with the hygromycin-phosphotransferase (*hph*) cassette, which was amplified from PHIG2RHPH2-GFP-GUS. The upstream and downstream flanking sequences were amplified with the 1F/2R and 3F/4R primers, respectively. After ligation with the *hph* cassette, the ligated PCR product was transformed into 03–8 according to the method described by Gao et al. (2011). Positive transformants were selected using hygromycin B at a final concentration of 100 μg/mL. The putative knockout mutants identified by screening with the primers 5F and 6R were further analyzed by PCR with the primers 7F and H855R and H856F and 8R to confirm the gene replacement events (Supplementary Figure S1). For the double gene knock-out mutants, the single gene knock-out mutant (Δ*VmDCL2*) was used to generate the protoplast, and another vector containing a geneticin resistance gene *NEO* was used to replace the ORF of the second gene. The detailed method used for the mutant generation is similar to that described above. The putative mutants were further confirmed by a Southern blot analysis with probes for the *HYG/NEO* fragments according to the manufacturer’s instructions of the DIG DNA Labeling and Detection Kit II (Roche, Mannheim, Germany). For the complementation assays, the *VmDCLs* were amplified using the primer pairs Com-F and Com-R. The resulting PCR products were cloned into the vector pFL2 using the yeast gap repair approach (Bruno et al., 2004; Zhou et al., 2011). The resulting fusion constructs rescued from the Trp^+^ yeast transformants were confirmed by sequencing and transformed into their respective *V. mali* deletion mutant. The geneticin-resistant transformants expressing the complementing constructs were identified by PCR using the primer pairs Com-F and Com-R. The primers used for the gene deletion and complementation are listed in Supplementary Table S1.

### Phenotypic Analysis of the *VmDCL* Mutants

To detect the growth rate of the colonies, *VmDCL* mutant strains (Δ*VmDCLs*) and 03–8 mycelium were placed onto the center of a PDA medium plate and were cultured in the dark at 25°C. The size of the colonies was examined after 24, 48, and 72 h. To observe the morphology of the colonies and hyphae, the colonies were photographed, and the hyphae morphology was observed using samples from the colony edge of each strain cultured in the dark at 25°C for 2 days under a microscope. To assay the stress response defects in the Δ*VmDCLs*, mycelium of the Δ*VmDCLs* and 03–8 were placed onto a PDA medium plate containing 0.05% H_2_O_2_ and 0.5 M KCl, and then, the plates were cultured in the dark at 25°C. The size and morphology of the colonies were examined after 3 days. The significance was analyzed using SPSS18 software. All experiments were performed in triplicate with three Petri dishes in each replication.

### Pathogenicity Test

‘Fuji’ apple (*Malus domestica* Borkh. cv ‘Fuji’) twigs were used to detect the pathogenicity of the Δ*VmDCLs* and 03–8. The test was conducted using the detailed methods described by Wei et al. (2010). The statistical significance of the differences was analyzed using SPSS18 software. The infection assays were repeated three times with six twigs per replicate.

### Library Preparation and RNA Deep Sequencing

Small RNA libraries were prepared following the detailed protocol provided by the genome sequencing company (Novogene, China). Three individual biological replicates of each sample (Δ*VmDCL1*, Δ*VmDCL2*, Δ*VmDCL1DCL2* and 03–8) were prepared, and the three sample replicates were mixed for the RNA isolation. For the small RNA library construction, three micrograms of total RNA from each sample were used as the input material. The sequencing libraries were generated using the NEBNext^®^ Multiplex Small RNA Library Prep Set for Illumina^®^ (NEB, United States) following the manufacturer’s recommendations, and index codes were added to attribute sequences to each sample. The library quality was assessed on an Agilent Bioanalyzer 2100 system using DNA High Sensitivity Chips. The clustering of the index-coded samples was performed on a cBot Cluster Generation System using the TruSeq SR Cluster Kit v3-cBot-HS (Illumia) according to the manufacturer’s instructions. After the cluster generation, the library preparations were sequenced on an Illumina HiSeq 2500/2000 platform, and 50 bp single-end reads were generated.

### Sequence Data Analysis

Raw data (raw reads) in the fastq format were first processed through custom Perl and python scripts. During this step, clean data (clean reads) were obtained by removing reads containing ploy-N, reads with 5′ adapter contaminants, reads without 3′ adapters or insert tags, reads containing ploy A, T, G or C, and low-quality reads from the raw data. The clean reads were mapped onto the reference sequence using Bowtie (Langmead et al., 2009) without a mismatch to confirm the sequence accuracy. The number of total unique sRNAs from the different samples was calculated to compare the difference among the Δ*VmDCLs* and 03–8. The common and specific sequences among the Δ*VmDCLs* and 03–8 were also compared. To preliminarily estimate the sRNAs varieties, the length distribution of each sample was also analyzed.

## Results

### Protein Sequence and Phylogenetic Tree Analysis

**FIGURE 1:**
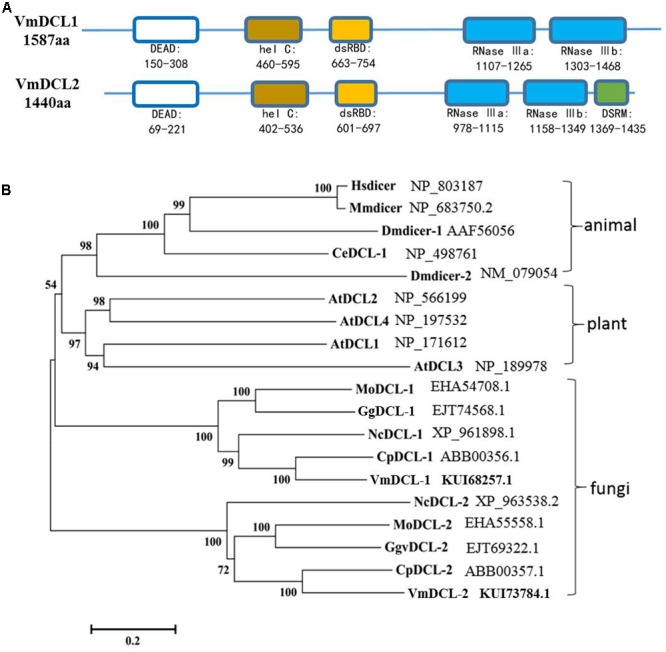
Domain structure and phylogeny of the VmDCLs. **(A)** Typical domains of the VmDCLs. VmDCL1 and VmDCL2 both contain five conserved domains, including DEAD, hel C, dsRBD, RNase IIIa and RNase IIIb. VmDCL2 also has a special domain, i.e., DSRM that is responsive to the generation of miRNAs. The number under the structures indicates the amino acid location of each domain. **(B)** Phylogenetic tree constructed via the multiple alignment program DNAMAN using DCLs sequences from plants, animals and fungi. VmDCLs clustered with other DCLs from fungi and shared a common ancestor with DCLs from plants and animals. Entries are labeled with the gene name in different species and the corresponding GenBank accession number. Hsdicer, Dicer of *Homo sapiens*, Mmdicer, Dicer of *Mus musculus*, Dmdicer, Dicer of *Drosophila melanogaster*, CeDCL, Dicer of *Caenorhabditis elegans*, AtDCL, Dicer of *Arabidopsis thaliana*, MoDCL, Dicer of *Magnaporthe oryzae*, GgDCL, Dicer of *Gaeumannomyces graminis* var. *tritici*, NcDCL, Dicer of *Neurospora crassa*, CpDCL, Dicer of *Cryphonectria parasitica*, VmDCL, Dicers of *Valsa mali*.

In total, there are two *DCL* homologous sequences in *V. mali*. The genes were designated *VmDCL1* and *VmDCL2*, and the sequences were deposited in GenBank with accession numbers KUI68257.1 and KUI73784.1, respectively. The InterProScan analyses showed that the deduced VmDCL1 protein contains five domains, which are known as RNA helicase domains (DEAD and hel C), dsRBD and Ribonuclease domains (RNase III a and RNase III b) and are characteristic of DCL proteins. Compared with VmDCL1, VmDCL2 also contains an additional special domain, i.e., DSRM (**Figure 1A**). In addition, the phylogenetic analysis suggests that the VmDCLs are clustered with the DCLs from fungi as expected, and they are close to the DCLs from *Cryphonectria parasitica* in evolution (**Figure 1B**).

### Acquisition of the *VmDCL* Mutants

**FIGURE 2:**
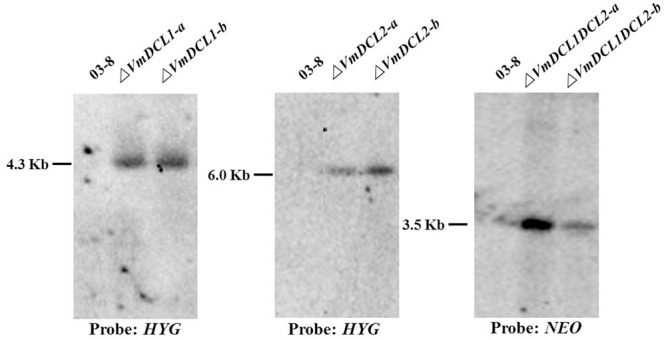
Construction of the *VmDCL* deletion mutants. Two candidate positive transformants of each mutant (Δ*VmDCL1-a* and Δ*VmDCL1-b*; Δ*VmDCL2-a* and Δ*VmDCL2-b*; and Δ*VmDCL1DCL2-a* and Δ*VmDCL1DCL2-b*) were used for Southern blotting. For Δ*VmDCL1*, genomic DNA from 03 to 8 and mutants was isolated and digested with restriction enzyme *Cla* I; for Δ*VmDCL2*, genomic DNA from 03 to 8 and Δ*VmDCL2* was isolated and digested with restriction enzyme *Sal* I; for Δ*VmDCL1DCL2*, genomic DNA from 03 to 8 and Δ*VmDCL2* was isolated and digested with restriction enzyme *Hind* III. DNA was hybridized with the probes *HYG* (Δ*VmDCL1* and Δ*VmDCL2*) and *NEO* (Δ*VmDCL1DCL2*).

As shown in Supplementary Figure S2, the *VmDCL1* single-gene deletion mutants (Δ*VmDCL1*), *VmDCL2* single-gene deletion mutants (Δ*VmDCL2*) and the *VmDCL1DCL2* double-gene deletion mutants (Δ*VmDCL1DCL2*) were identified via screening with different PCR primers. The results indicated that we obtained putative knockout mutants of *VmDCL1, VmDCL2* and *VmDCL1DCL2*. Subsequently, all putative knockout mutants were further verified by Southern blot hybridization. Furthermore, no hybridization signal was detected in the wild type strain 03–8 using the *HYG* probe amplified with the primers HYG/F and HYG/R, but there was a 4.3 and 6.0 Kb band in the *VmDCL1* and *VmDCL2* mutants, respectively; meanwhile, a *NEO* probe amplified with the primers NEO/F and NEO/R was used to hybridize with the genomic DNA of Δ*VmDCL1DCL2*, and a single corresponding 3.5 Kb band was detected. Thus, the single copy mutants of *VmDCL1, VmDCL2* and *VmDCL1DCL2* were generated successfully (**Figure 2**).

### Growth Rate and Morphology of the Colony and Hypha Showed No Obvious Change in the Mutants Compared with Those in 03–8

To investigate whether *VmDCLs* play a critical function in the growth and development of *V. mali*, the growth rate and morphology of the colony and hypha of the mutants (Δ*VmDCL1*, Δ*VmDCL2* and Δ*VmDCL1DCL2*) were compared with those of 03–8. No obvious difference in the colonial morphology was observed between the mutants and 03–8 when they were cultured on a PDA medium at 25°C in the dark for 2 days (Supplementary Figure S3a). Furthermore, the hypha morphology did not change obviously (Supplementary Figure S3b). In addition, the growth rate of 03–8, Δ*VmDCL1*, Δ*VmDCL2* and Δ*VmDCL1DCL2* was 2.50, 2.48, 2.52, and 2.47 cm/d, respectively, also showing no statistically significant difference (Supplementary Figure S3c).

### *VmDCLs* Were Involved in the Stress Responses

**FIGURE 3:**
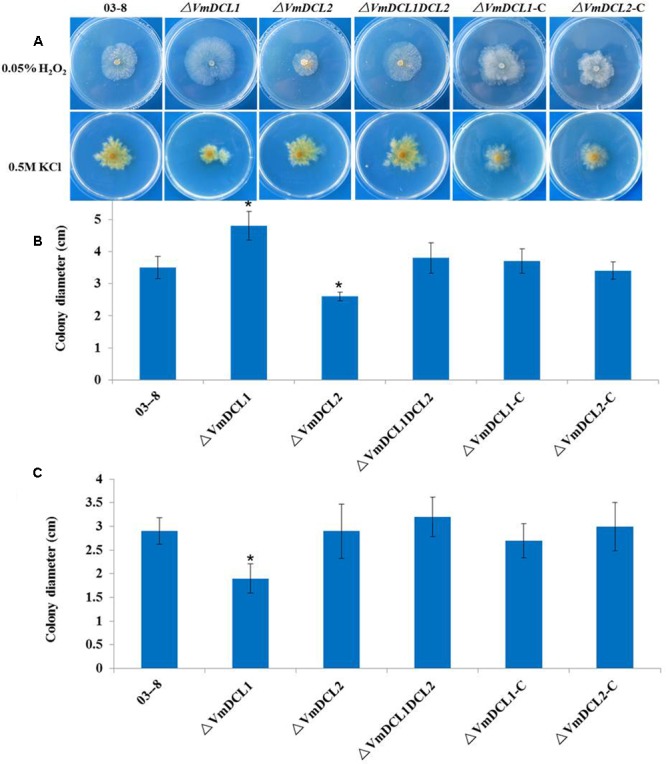
Abiotic stress responses of *VmDCL* mutants and 03–8. 03–8, Δ*VmDCL1*, Δ*VmDCL2*, Δ*VmDCL1DCL2* and the complementary mutants (Δ*VmDCL1*-C *and*Δ*VmDCL2*-C) were cultured on PDA supplemented with 0.05% H_2_O_2_ **(A,B)** and 0.5 M KCl **(A,C)**. Photographs were taken after 3 days of incubation at 25°C. All experiments were performed in triplicate in three Petri dishes in each replication. The bars indicate the standard deviation of the mean of three replicates. The stars above the bars indicate significantly different means (LSD test, *P* < 0.05).

To determine whether *VmDCLs* were involved in the stress responses, H_2_O_2_ and KCl were added to the PDA medium. In the presence of 0.05% H_2_O_2_, Δ*VmDCL2* grew more slowly than 03–8 3 days after the inoculation, but the colony of Δ*VmDCL1* was much larger than the colony of 03–8. Interestingly, the colony of Δ*VmDCL1DCL2* showed no obvious change compared with that of 03–8 (**Figures 3A,B**). In the presence of 0.5 M KCl, the colony growth of only Δ*VmDCL1* was significantly inhibited, while there was no significant difference among 03–8, Δ*VmDCL2* and Δ*VmDCL1DCL2* (**Figures 3A,C**). The complemented strain showed a nearly normal growth compared with 03–8 under the H_2_O_2_ and KCl treatment conditions. These results suggest that the *VmDCLs* may be important but play distinct roles in the response to oxidative and osmotic stresses.

### *VmDCLs* Play Distinct Roles in the Pathogenicity of *V. mali*

**FIGURE 4:**
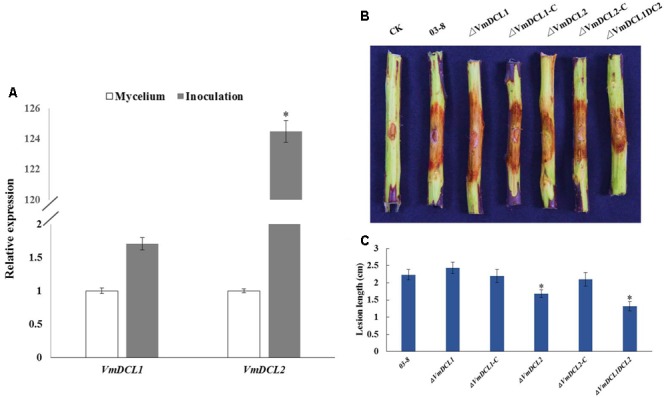
Phenotypical analysis of *VmDCL* mutants and 03–8. **(A)** Transcript levels of *VmDCL1* and *VmDCL2* at 48 hpi compared to those of the control (mycelium) using *G6PDH* for normalization. The results were calculated using the comparative threshold (2^-ΔΔCT^) method. Vertical bars represent the standard deviation. The mean values and standard deviations were calculated using data from three independent replicates. **(B)** ‘Fuji’ apple (*Malus domestica* Borkh. cv ‘Fuji’) twigs were used to determine the pathogenicity of the mutants relative to that of 03–8. Photographs were taken at 3 days after inoculation. **(C)** Lesion diameters of twigs were measured 3 days after the inoculation. The bars indicate the standard deviation of the mean of three biological replicates. The stars above the bars indicate significantly different means (LSD test, *P* < 0.05).

The expression analysis of the *VmDCLs* during the *V. mali* infection in apple twig showed that compared with the control (mycelium), *VmDCL2* was up-regulated and peaked (125-fold) at 48 hpi. However, *VmDCL1* expressed stably with no significant change and only exhibited a 1.7-fold increase at 48 hpi (**Figure 4A**). The pathogenicity of Δ*VmDCLs* and 03–8 was further analyzed. Compared with 03–8, the pathogenicity of Δ*VmDCL1* showed no significant change, while the Δ*VmDCL2* was defective in plant infections with a smaller lesion. Furthermore, the pathogenicity markedly decreased when both *VmDCL1* and *VmDCL2* were knocked out together. Under the same conditions, the pathogenicity of the complemented transformants Δ*VmDCL1*-C and Δ*VmDCL2*-C was similar to that of 03–8 in the twig infection assays (**Figures 4B,C**). These results suggest that *VmDCL2* plays a critical role during the infection process in twigs, and the detailed mechanism will be a key scientific problem to be explored in the future.

### *VmDCLs* Were Associated with sRNA Generation

**FIGURE 5:**
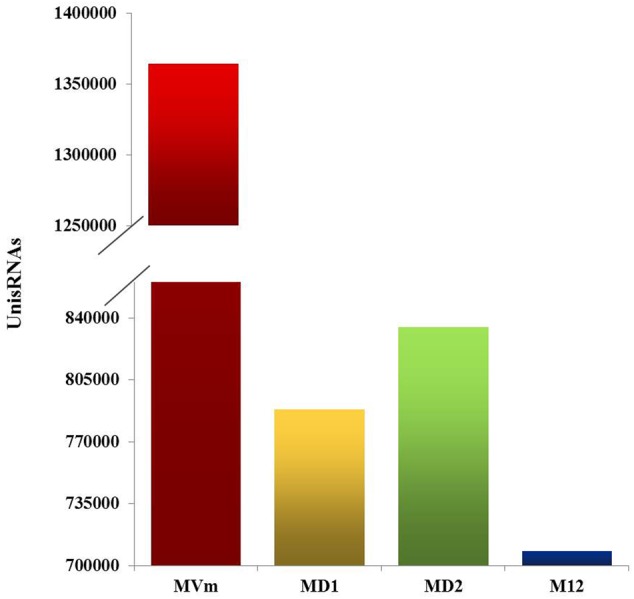
Quantification of the unisRNAs quantity in the *VmDCL* mutants and 03–8. Total number of uniRNAs in 03–8 and each mutants was analyzed, respectively. MVm, 03–8; MD1, Δ*VmDCL1*; MD2, Δ*VmDCL2*; MD12, Δ*VmDCL1DCL2.*

**FIGURE 6:**
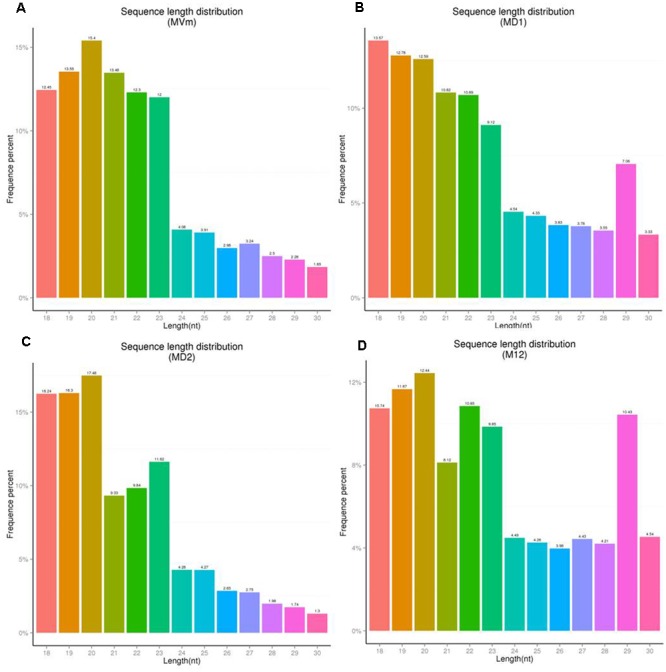
Sequence length distribution of unisRNAs in the *VmDCL* mutants and 03–8. The length distribution of the unisRNAs changed when *VmDCL1* **(B)**, Δ*VmDCL2* **(C)** and Δ*VmDCL1DCL2* **(D)** were deleted compared with 03–8 **(A)**. Horizontal axis indicates the sequence length, the vertical axis indicates the proportion of the corresponding reads, and the colors indicate the different lengths. MVm, 03–8; MD1, Δ*VmDCL1*; MD2, Δ*VmDCL2*; MD12, Δ*VmDCL1DCL2.*

To comprehensively examine the sRNA abundance in strains of 03–8 (MVM), Δ*VmDCL1* (MD1), Δ*VmDCL2* (MD2) and Δ*VmDCL1DCL2* (MD12), four sRNA libraries were constructed and sequenced using an Illumina Analyzer. The distinct libraries generated 12818591, 6927296, 11565746 and 8990152 raw reads. After removing the low-quality and non-sense sequences (<15 nt), in total, 1364324, 788373, 834923 and 708262 unisRNAs were isolated (data not shown). As shown in **Figure 5**, the number of unisRNAs was markedly smaller in the libraries of MD1, MD2 and MD12 than that in the library of MVM, indicating that the deletion of the *VmDCLs* could affect the generation of sRNAs in *V. mali*. In addition, the length distribution of the unisRNAs was further analyzed (18–30 nt long), and the length distribution also markedly changed when the *VmDCLs* were deleted. When *VmDCL1* was knocked out, the abundance of sRNAs consisting of 29 nt showed the most obvious increase (**Figures 6A,B**). Meanwhile, the abundance of sRNAs consisting of 18–20 nt noticeably increased, and the abundance of sRNAs consisting of 21–22 nt noticeably decreased when *VmDCL2* was knocked out (**Figures 6A,C**). Additionally, in the Δ*VmDCL1DCL2*, the abundance of sRNAs consisting of 21 nt noticeably decreased, and the abundance of sRNAs consisting of 29 nt noticeably increased (**Figures 6A,D**). The other sRNA abundances were also affected in varying degrees between the mutants and 03–8. The results indicated that the *VmDCLs* could affect the generation of sRNAs in *V. mali*, and their function is likely redundant. Furthermore, there are at least two pathways (i.e., DCL-dependent and DCL-independent) for sRNA generation in *V. mali*. The detailed mechanism will be explored in further studies.

## Discussion

RNAi, which is mediated by small regulatory RNAs, plays important roles in various biological progresses at the transcriptional and post-transcriptional levels (Pratt and MacRae, 2009). In filamentous fungi, several unique classes of sRNAs have been identified, particularly in *N. crassa*, although the corresponding targets remain to be fully elucidated (Lee et al., 2009, 2010). In other fungal species, particularly in plant pathogenic fungi, small RNA-mediated gene silencing remains in its initial exploration phase. As increasing numbers of fungal genomes were published and deep sequence technologies and bioinformatics analyses continuously advanced, more fungi were confirmed to possess the RNA silencing mechanism (Nunes et al., 2011a; Zhou J. et al., 2012; Zhou Q. et al., 2012; Kang et al., 2013; Lau et al., 2013; Campo et al., 2016). Information regarding the genome of *V. mali*, which is an important weak parasitic plant pathogen, has become public, and the major components of the RNAi pathway were also predicted (Yin et al., 2015). However, little is known regarding the function of RNAi pathway in *V. mali*.

In this study, the ‘switch’ for the generation and action of sRNAs, i.e., Dicer-like genes, in *V. mali* were isolated and functionally analyzed. Based on the known genome information of *V. mali*, two DCL genes (*VmDCL1* and *VmDCL2*) were isolated, and the VmDCLs were grouped clearly with DCL members from other fungal species assessed by the phylogenetic analysis. Most of the fungi have two DCL-like proteins, particularly in Ascomycota (Nunes et al., 2011b). In addition, both VmDCL1 and VmDCL2 contain an RNA helicase domain, RNase III domains and double-stranded RNA binding domain, which are the typical domains of DCL proteins (MacRae et al., 2006). However, neither DCL in *V. mali* contains a PAZ (Piwi/Argonaute/Zwille) domain. Some eukaryotes have been found to lack the PAZ domain, such as *Colletotrichum higginsianum*, but they still retain the capacity to generate small RNAs (Casas-Mollano et al., 2008; Drinnenberg et al., 2009; Braun et al., 2010; Campo et al., 2016). Furthermore, VmDCL2 contains an additional double-stranded RNA binding motif (DSRM). The DSRM is an important domain of DGCR8, which is required for microRNA (miRNA) processing in the RNAi pathway (Denli et al., 2004; Yeom et al., 2006). Therefore, VmDCL2 may be closely involved in the generation of miRNAs.

The biological function of DCLs in different species is diverse. In previous studies, disruptions of DCL orthologs were sufficient to slow vegetative growth in *M. circinelloides, M. oryzae, B. cinerea* and *T. atroviride* (Kadotani et al., 2004; Nicolás et al., 2007; Carreras-Villaseñor et al., 2013; Weiberg et al., 2013). However, the deletion of *DCL* did not affect the growth of *Cryphonectria parasitica* and *Saccharomyces castellii* (Segers et al., 2007; Drinnenberg et al., 2009). To evaluate the function of *VmDCLs*, mycelial growth and morphology on a PDA medium were measured based on the generation of Δ*VmDCL1*, Δ*VmDCL2* and Δ*VmDCL1DCL2*. The colony and hypha morphology and mycelial growth were indistinguishable between the mutants and the wild type, suggesting that the *VmDCLs* are not necessary for vegetative growth. Moreover, Δ*VmDCL1* showed an increased adaptability to 0.05% H_2_O_2_ and a sensibility to 0.5 M KCl, while Δ*VmDCL2* only exhibited an increased sensibility to 0.05% H_2_O_2_. The similar phenotype changes were also confirmed in the *dcr1, dcr2* and *dcr1dcr2* mutants of *T. atroviride*, which suggested that each DCL controls different biological processes (Carreras-Villaseñor et al., 2013). The *VmDCL*s play an important but distinct role in the pathogenicity of *V. mali*. Using qRT-PCR, we found that *VmDCL2* was up-regulated significantly during the infection process. Furthermore, we found that the deletion of *VmDCL2* and *VmDCL1DCL2* resulted in a significant reduction in pathogenicity. Thus, we speculated that *VmDCL2* is a critical factor in the pathogenicity of *V. mali*, and the sRNAs generated by *VmDCL2* may be responsive to the pathogenicity. In *M. oryzae*, no DCLs mutants showed obvious phenotypes associated with pathogenicity (Kadotani et al., 2004). However, the *dcl1dcl2* double-mutant of *B. cinerea* showed a weakened pathogenicity regulated by an sRNA (Weiberg et al., 2013). These results also indicated that the functions of the DCLs were diversified in the evolution of different fungi.

To investigate the function of *VmDCLs* for sRNA generation, we compared the sRNA amounts in mutants and wild type based on high-throughput sequencing. Based on the results, the total sRNA amount was reduced greatly and the number of sRNAs with 21 and 29 nt changed when *VmDCL1, VmDCL2* and *VmDCL1DCL2* were knocked out. This finding indicated that *VmDCLs* were related to sRNA biogenesis. However, except for the specific unisRNAs, there were still many common sRNAs in both the wild type and Δ*VmDCL1/*Δ*VmDCL2*. DCLs appear to also be functionally redundant in sRNA processing in certain fungi species (Lee et al., 2010; Nicolás et al., 2013; Weiberg et al., 2013). We also found a slew of new sRNAs that could be generated in the *VmDCL* deletion mutants, and the length distribution of these sRNAs was also different than that in the wild type. This finding confirmed that the function of the *VmDCL*s in sRNA generation is redundant, and there is more than one pathway responsible for sRNA biogenesis. A new pathway could be activated when the *VmDCLs* were deleted. In previous studies, at least four different biogenesis pathways of miRNA-like RNAs have been identified in *N. crassa*, including both DCL-dependent and DCL-independent pathways (Jin and Zhu, 2010; Lee et al., 2010; Li et al., 2010). Thus, whether the phenotypical changes in the *VmDCL* deletion mutants were associated with the generation of different sRNAs is an interesting scientific question that should be explored.

In summary, we isolated and identified DCLs from *V. mali* for the first time and investigated their gene function in development, abiotic stress response, and pathogenicity. Another major finding of this study is the functional confirmation of the *VmDCLs* in the generation of sRNAs. In addition, the possible existence of diverse sRNA biogenesis pathways in *V. mali* presents a research direction for the identification of sRNA species and the characterization of regulatory sRNAs in pathogenicity.

## Author Contributions

HF and LH were responsible for the experimental design, and provided guidance on the whole study. HF wrote the manuscript and LH further revised it. HF, MX, YL, and RD constructed the mutant strains and determined the phenotypes. XG prepared cDNA samples and detected the gene expression. All authors have read and approved the manuscript.

## Conflict of Interest Statement

The authors declare that the research was conducted in the absence of any commercial or financial relationships that could be construed as a potential conflict of interest.

## Footnotes

1 http://www.ebi.ac.uk/interpro/search/sequence-search
