# Corrigendum: Dicer-Like Genes Are Required for H_2_O_2_ and KCl Stress Responses, Pathogenicity and Small RNA Generation in *Valsa mali*

**DOI:** 10.3389/fmicb.2017.01457

**Published:** 2017-07-28

**Authors:** Hao Feng, Ming Xu, Yangyang Liu, Ruqing Dong, Xiaoning Gao, Lili Huang

**Affiliations:** College of Plant Protection and State Key Laboratory of Crop Stress Biology for Arid Areas, Northwest A&F University Yangling, China

**Keywords:** dicer, pathogenesis, RNAi mechanism, small RNA, *Valsa mali*

In the original article it was not indicated that Ming Xu was a joint first author. The authors apologize for this error and state that this does not change the scientific conclusions of the article in any way.

## Conflict of interest statement

The authors declare that the research was conducted in the absence of any commercial or financial relationships that could be construed as a potential conflict of interest.

