# Supplementary material for: Dicer-Like Genes Are Required for H2O2 and KCl Stress Responses, Pathogenicity and Small RNA Generation in Valsa mali
Source: Front Microbiol. 2017 Jun 23;8:1166. doi: 10.3389/fmicb.2017.01166 (PMC5481355; doi:10.3389/fmicb.2017.01166)
Supplement: Supplementary file 1 [file Table_1.doc]

Table S1. Primers used in vector construction and transformants detection

| Names | Primer sequences (5' to 3') |
| --- | --- |
| VMDCL1/1F | AGCTGAGGACATCGCGGTAG |
| VMDCL1/2R | TTGACCTCCACTAGCTCCAGCCAAGCCGGGCAAGGAATGGGAAGG |
| VMDCL1/3F | GAATAGAGTAGATGCCGACCGCGGGTTTACGAGTCCCTTTGCTTGATG |
| VMDCL1/4R | AGTTGGCGGGTGGATGAG |
| VMDCL1/CF | GGTTCGTGCTGAGTTGCG |
| VMDCL1/CR | AGTTGGCGGGTGGATGAG |
| VMDCL1/5F | GCTCTGCCACCAGGACGACT |
| VMDCL1/6R | TCCCACCACAACAGGTTGCTTC |
| VMDCL1/7F | GCTCTTCGCTGTATGTCTCG |
| VMDCL1/8R | CGTCATCGTATGGTGGGTC |
| VMDCL2/1F | GCTTGGGATTGGTGGTATTG |
| VMDCL2/2R | TTGACCTCCACTAGCTCCAGCCAAGCCACACTGCTGTCGTGGCTCTT |
| VMDCL2/3F | GAATAGAGTAGATGCCGACCGCGGGTTTTGAAGATTGAAGAAAGGGGG |
| VMDCL2/4R | AAGGCTGTCACAAATGAGGGT |
| VMDCL2/CF | TTGGTGGTATTGTTGGACTTCA |
| VMDCL2/CR | AAATGAGGGTGAGACTTCGTTT |
| VMDCL2/5F | CCACATTGGATGCCTACTGTCG |
| VMDCL2/6R | CCAGAACATTCCACTCCTTTCG |
| VMDCL2/7F | CGAGGACTACCTTACGAACCTA |
| VMDCL2/8R | CCAACCTTGCTAACGGAGAA |
| dVMDCL1/2R | CAGATACGGCAGAGAAATCGCAACCTCGGGCAAGGAATGGGAAGG |
| dVMDCL1/3F | GTTTAGATTCCAAGTGTCTACTGCTGGCTACGAGTCCCTTTGCTTGATG |
| qDCL1/F | CGGCGCAAAGATCACCTA |
| qDCL1/R | CTACCTCGCATTGGAACCC |
| qDCL2/F | GGCAAGAAATACGGCAAAC |
| qDCL2/R | CGAAGAGGTCCGAGAAGAAC |
| HYG/F | GGCTTGGCTGGAGCTAGTGGAGGTCAA |
| HYG/R | AACCCGCGGTCGGCATCTACTCTATTC |
| NEO/F | GAGGTTGCGATTTCTCTGCCGTATCTG |
| NEO/R | GCCAGCAGTAGACACTTGGAATCTAAAC |
| H855R | GCTGATCTGACCAGTTGC |
| H856F | GTCGATGCGACGCAATCGT |
| H852F | ATGTTGGCGACCTCGTATTGG |
| H850R | TTCCTCCCTTTATTTCAGATTCAA |
| G855R | TGTTGGGTTTGAGCTAGGTGGG |
| G856F | GAATGGTCAAATCAAACTGCTAGATAT |
| G852F | TCGGCTATGACTGGGCACAACA |
| G850R | GAGCGGCGATACCGTAAAGCAC |
| VMDCL1-CM-F | cgactcactatagggcgaattgggtactcaaattggGGTGTGGACCTTCTGGATGA |
| VMDCL1-CM-R | caccaccccggtgaacagctcctcgcccttgctcacAATAGCCGTCGCGCTCTC |
| VMDCL2-CM-F | cgactcactatagggcgaattgggtactcaaattggCCTAGACAGGCGGCGTAC |
| VMDCL2-CM-R | caccaccccggtgaacagctcctcgcccttgctcacAGGGCCCCTACCAGACAACA |
