# Supplementary figures and images for: Dicer-Like Genes Are Required for H2O2 and KCl Stress Responses, Pathogenicity and Small RNA Generation in Valsa mali
Source: Front Microbiol. 2017 Jun 23;8:1166. doi: 10.3389/fmicb.2017.01166 (PMC5481355; doi:10.3389/fmicb.2017.01166)

Fig. S1 Map for Double-joint PCR

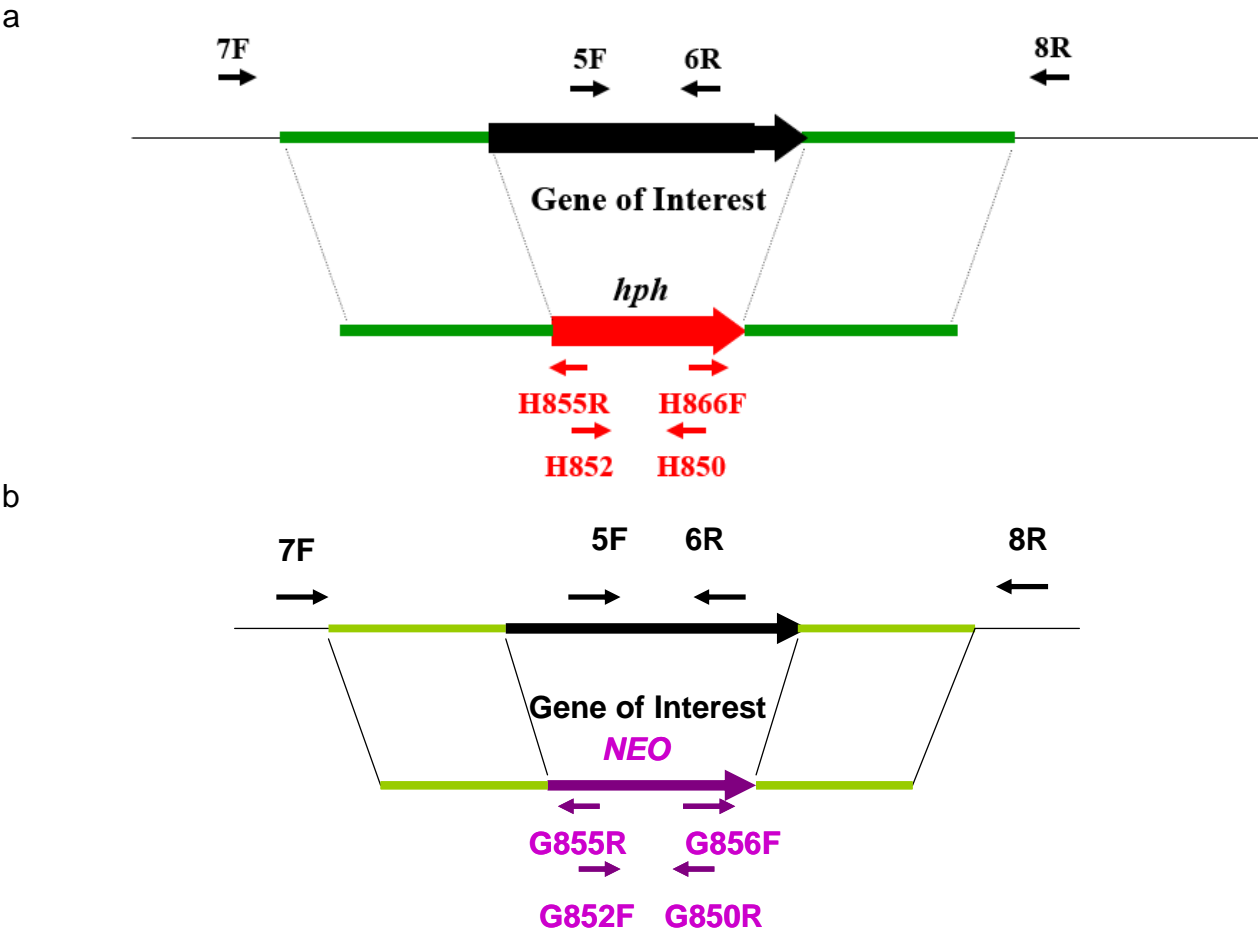

Supplement: Supplementary file 2 [file Image_1.PDF]
