# Supplementary material for: Dicer-Like Genes Are Required for H2O2 and KCl Stress Responses, Pathogenicity and Small RNA Generation in Valsa mali
Source: Front Microbiol. 2017 Jun 23;8:1166. doi: 10.3389/fmicb.2017.01166 (PMC5481355; doi:10.3389/fmicb.2017.01166)
Supplement: Supplementary file 3 [file Image_2.PDF]

Fig. S2 PCR detection of *VmDCLs* deletion mutants using four pairs of primers.

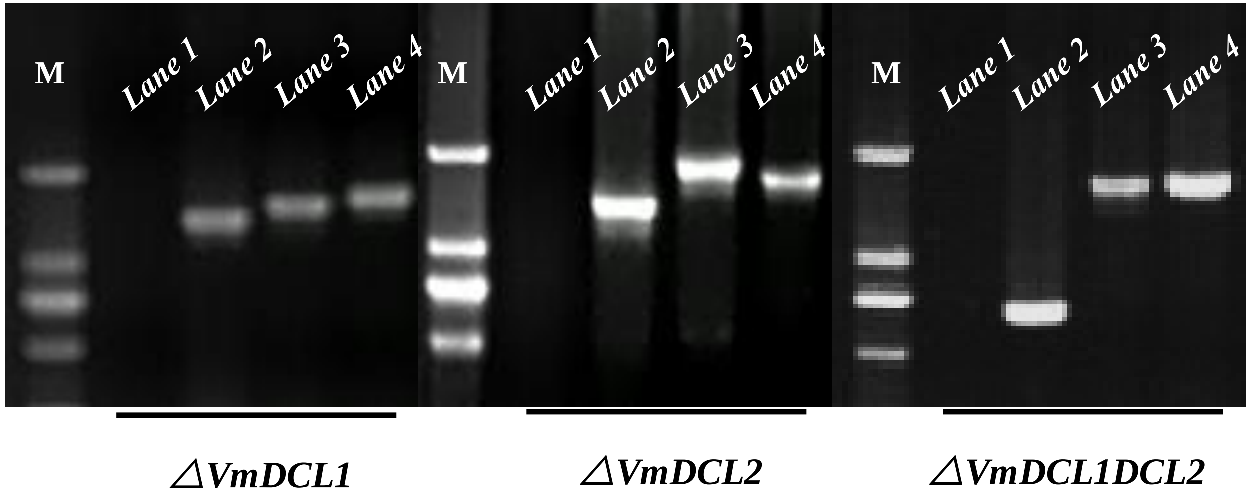

The protoplast of wild type 03-8 was used for *VmDCL1* and *VmDCL2* single gene deletion mutants (△*VmDCL1* and △*VmDCL2*). M: DM2000 maker. For △*VmDCL1*detection, Lane 1: product amplified by VmDCL1-5F/VmDCL1-6R for detecting *VmDCL1*. Lane 2: product amplified by H852/H850 for detection of the *HYG* insertion. Lane 3 and lane 4: products amplified by VmDCL1-7F /H855R and H856F/ VmDCL1-8R for confirming homologous recombination. For △*VmDCL2* detection, Lane 1: product amplified by VmDCL2-5F/VmDCL2-6R for detecting *VmDCL2*. Lane 2: product amplified by H852/H850 for detection of the *HYG* insertion. Lane 3 and lane 4: products amplified by VmDCL2-7F/H855R and H856F/VmDCL2-8R for confirming homologous recombination. The protoplast of △*VmDCL1* was used for *VmDCL1* and *VmDCL2* double genes deletion mutant (△*VmDCL1DCL2*). For △*VmDCL1DCL2* detection, Lane 1: product amplified by VmDCL2-5F/VmDCL2-6R for detecting *VmDCL2*. Lane 2: product amplified by G850/ G852 for detection of the *NEO* insertion. Lane 3 and lane 4: products amplified by VmDCL2-7F/G855R and G856F/VmDCL2-8R for confirming homologous recombination.
