# Supplementary material for: Dicer-Like Genes Are Required for H2O2 and KCl Stress Responses, Pathogenicity and Small RNA Generation in Valsa mali
Source: Front Microbiol. 2017 Jun 23;8:1166. doi: 10.3389/fmicb.2017.01166 (PMC5481355; doi:10.3389/fmicb.2017.01166)
Supplement: Supplementary file 4 [file Image_3.PDF]

Fig. S3 Vegetable growth between *VmDCLs* mutants and 03-8

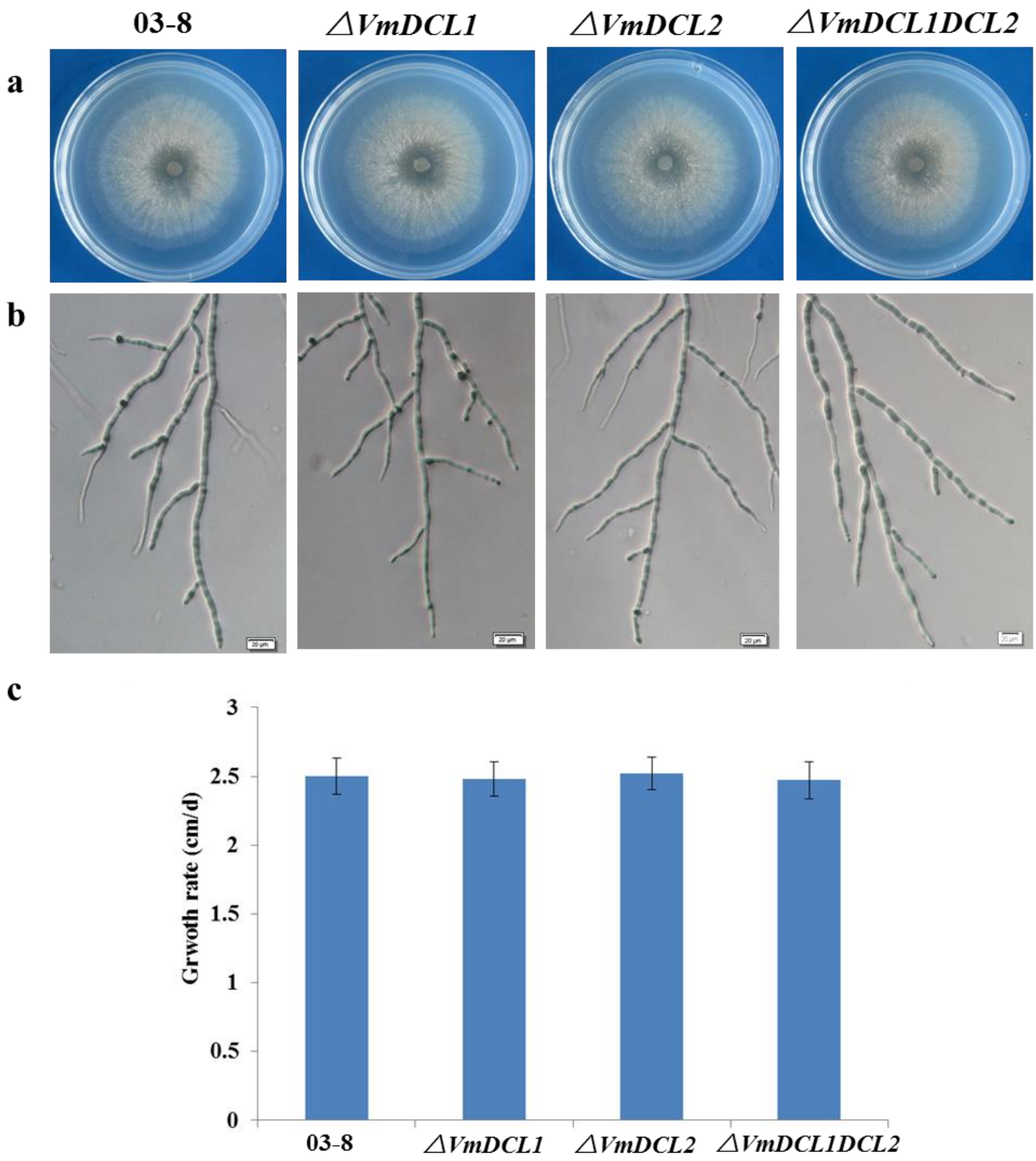

a. Colony morphology of *VmDCL1*, *VmDCL2* and *VmDCL12* gene knock-out mutants (PDA, 25℃, 2d). **b.** Hypha morphology of *VmDCL1*, *VmDCL2* and *VmDCL12* gene knock-out mutants (PDA, 25℃, 2d). **c.** Growth rate of *VmDCL1*, *VmDCL2* and *VmDCL12* gene knock-out mutants (PDA, 25℃). All experiments were performed in triplicate, with three petri dishes in each repetition.
